# Pre-hospital management of mass casualty civilian shootings: a systematic literature review

**DOI:** 10.1186/s13054-016-1543-7

**Published:** 2016-11-08

**Authors:** Conor D. A. Turner, David J. Lockey, Marius Rehn

**Affiliations:** 1 Barts and The London School of Medicine and Dentistry, Queen Mary University London, Garrod Building, Turner Street, Whitechapel, London, E1 2AD UK; 2 London’s Air Ambulance, Barts Health Trust, London, UK; 3 The Norwegian Air Ambulance Foundation, Drøbak, Norway; 4 Department of Health Studies, University of Stavanger, Stavanger, Norway

**Keywords:** Pre-hospital management, Mass shootings, Civilian, Haemorrhage control, Triage, Tactical Emergency Medical Services

## Abstract

**Background:**

Mass casualty civilian shootings present an uncommon but recurring challenge to emergency services around the world and produce unique management demands. On the background of a rising threat of transnational terrorism worldwide, emergency response strategies are of critical importance. This study aims to systematically identify, describe and appraise the quality of indexed and non-indexed literature on the pre-hospital management of modern civilian mass shootings to guide future practice.

**Methods:**

Systematic literature searches of PubMed, Cochrane Database of Systematic Reviews and Scopus were conducted in conjunction with simple searches of non-indexed databases; Web of Science, OpenDOAR and Evidence Search. The searches were last carried out on 20 April 2016 and only identified those papers published after the 1 January 1980. Included documents had to contain descriptions, discussions or experiences of the pre-hospital management of civilian mass shootings.

**Results:**

From the 494 identified manuscripts, 73 were selected on abstract and title and after full text reading 47 were selected for inclusion in analysis. The search yielded reports of 17 mass shooting events, the majority from the USA with additions from France, Norway, the UK and Kenya. Between 1994 and 2015 the shooting of 1649 people with 578 deaths at 17 separate events are described. Quality appraisal demonstrated considerable heterogeneity in reporting and revealed limited data on mass shootings globally.

**Conclusion:**

Key themes were identified to improve future practice: tactical emergency medical support may harmonise inner cordon interventions, a need for inter-service education on effective haemorrhage control, the value of senior triage operators and the need for regular mass casualty incident simulation.

## Background

Few man-made or natural catastrophes are as unsettling as intentional mass murder. Perhaps most disturbing of all is the indiscriminate attack of a “lone wolf”, or “active shooter” gunmen on innocent civilians.

“Mass shooting” is a phrase that is propagated by media outlets, government papers and occasionally scientific journals, and yet a universal definition does not exist. The Federal Bureau of Intelligence (FBI) describes mass killings as the killing of three or four or more persons without an extended interruption. The FBI also defines “active shooters” as “an individual actively engaged in killing or attempting to kill people in a confined and populated area” [1]. The US congressional research service report on public mass shootings describes it as an incident in a public place, killing four or more people in an indiscriminate manner [2]. A simple definition is helpful from a medical perspective, as emergency services are usually mobilised without comprehensive information.

Motives of mass murderers include politics, revenge, hate, perverted love, and premeditated execution [3]. A strong link between mass murder and mental illness has not been established [4].

Major incident is defined in a consensus paper on reporting pre-hospital major incident medical management as “an incident that requires the mobilization of extraordinary Emergency Medical Services (EMS) resources and is identified as a major incident in that system” [5]. Inclusion of only civilian major incidents allows comparison of civilian pre-hospital medical services and avoids confusion with very differently prepared and resourced military medical services. For the purpose of this review a “mass shooting” will refer to a civilian major incident caused by the actions of an “active shooter”.

Given the rising threat of transnational terrorism, emergency response strategies are of critical importance [6]. There is a need to provide useful evidence and experience-based information for those who may become involved in the response to future active-shooter major incidents. This systematic review aims to identify, describe and appraise the quality of indexed and non-indexed literature on the pre-hospital management of modern civilian mass shootings to guide future practice.

## Methods

### Search strategy

A systematic literature search was undertaken on PubMed, Cochrane Database of Systematic Reviews and Scopus for the period of 1 January 1980 to the 20 April 2016. Medical Subject Headings terms were combined with non-indexed relevant search words to create a set of sensitive entry criteria (Table 1). Second, a systematic search of the grey literature was conducted using the Luxembourg Definition for grey literature [7]. The same entry criteria displayed in Table 1 as for the published literature were used for Web of Science, and simple search criteria were used for OpenDOAR and Evidence Search. The search was conducted over the same time period. The references from included papers were checked for additional material not found on the original search.

**Table 1:** Systematic search strategy

	Search terms
1. Set of entry criteria	((((((mass casualty incident) OR major incident) OR multiple casualty incident) OR mass casualty event) OR terrorism) OR terrorist attack))
2. Set of entry criteria	((((mass shooting) OR shooting) OR gunshot) OR firearm) OR active shooter)
3. Set of entry crtieria	((((pre hospital management) OR pre hospital care) OR emergency medical services) OR emergency medical management)
4. Final search	1 AND 2 AND 3

### Selection criteria

For the inclusion criteria the manuscripts had to contain descriptions, discussions or experiences of the pre-hospital management of civilian mass shootings as defined in the introduction, and had to be written in English, published between the 1 January 1980 and 20 April 2016 and be relevant to pre-hospital immediate medical management. Manuscripts were excluded if they only discussed mass casualty incidents (MCIs) due to blast injury.

One author (CDAT) screened the titles or abstracts of identified literature. Literature clearly not complying with the inclusion criteria was excluded. Eligibility was assessed by full text reading of the uncertain papers and inclusion was subject to consensus with a second author (MR). The Preferred Reporting Items for Systematic Reviews and Meta-Analyses (PRISMA) guideline was followed [8].

### Data extraction and quality appraisal

Data extraction focused on identifying common themes in the case reports and other manuscripts, recording information under ten agreed questions on a Microsoft Excel Spreadsheet Version 14.5.1 (2011; Microsoft Corporation, USA) Quality was appraised using a predefined checklist of questions depicting internal and external validity and were evaluated against the Oxford Centre for Evidenced-based Medicine (CEBM) Levels of Evidence [9].

## Results

### Identification

**Fig. 1:**
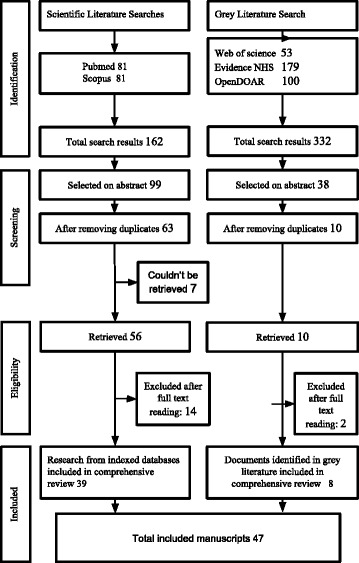
Flow chart depicting the stages of the systematic literature search. *NHS* National Health Service

Among the 494 identified manuscripts, 73 were selected on abstract and title and after full text reading 47 were selected for inclusion in analysis (Fig. 1). Seven papers could not be retrieved.

### Quality appraisal

Study design in the included manuscripts comprised case reports (*n* = 14), review articles (*n* = 4), commentaries (*n* = 14), government reports (*n* = 5), information technology report (*n* = 1), consensus statements (*n* = 2) and original research papers on matters relevant to pre-hospital management of mass casualty civilian shootings (*n* = 7) [1, 2, 10–54]. The search revealed heterogeneous primary source information on seventeen mass shooting events, the majority from the USA and others from Norway, the UK, Kenya and France (Table 2). According to the Oxford UK CEBM Levels of Evidence the literature was of levels 5 (*n* = 27), 4 (*n* = 18) and 3b (*n* = 2).

**Table 2:** Quality appraisal of primary sources using predefined checklist

Civilian Mass Shooting	Fair Child base	Dunblane School	Columbine School	West Nickles Mines	Virginia Tech		Mumbai	Fort Hood Base 1	Cumbria	Utoya Island		Tuscon	Dark Knight Cinema			Minneapolis Work Place	Westgate Shopping Centre	Columbia Mall	Fort Hood Base 2	Charlie Hebdo	Paris attacks		
Author	Beyersdorf et al.	Cullen et al.	Mell et al.	Ressel et al.	Kaplowitz et al.	Timothy et al.	Roy et al.	Wild et al.	Chesterman et al.	Sollid et al.	Waage et al.	Barr et al.	Lindique et al.	Johnson et al.	Koehler et al.	Autrey et al.	Wachira et al.	Levy et al.	Strommen et al.	Tourtier et al.	Hirsch et al.	Phillipe et al.	Lawrence et al.
Year of shooting	1994	1996	1999	2006	2007	2007	2008	2009	2010	2011	2011	2011	2012	2012	2012	2012	2013	2014	2014	2015	2015	2015	2015
1. Number of studies describing the incidents?	1	1	1	1	2		1	1	1	2		1	3			1	1	1	1	1	3		
2. Literature indexed?	**✔**	**✕**	**✔**	**✔**	**✔**	**✕**	**✔**	**✔**	**✕**	**✔**	**✔**	**✔**	**✔**	**✔**	**✔**	**✔**	**✔**	**✔**	**✔**	**✔**	**✔**	**✔**	**✔**
3. Authors involved in medical response?	**?**	**✔**	**?**	**?**	**?**	**?**	**?**	**?**	**✔**	**✔**	**✔**	**?**	**✔**	**✔**	**?**	**?**	**✔**	**✔**	**?**	**?**	**✔**	**?**	**?**
4. Reference to data sources provided?	**✔**	**✔**	**✔**	**✕**	**✔**	**✔**	**✔**	**✔**	**✔**	**✔**	**✔**	**✕**	**✕**	**✕**	**✕**	**✔**	**✔**	**✕**	**✕**	**✕**	**✔**	**✕**	**✕**
5. Conflict of interests depicted?	**✕**	**✔**	**✕**	**✕**	**✕**	**✔**	**✔**	**✔**	**✔**	**✔**	**✔**	**✕**	**✕**	**✕**	**✕**	**✔**	**✔**	**✔**	**✔**	**✕**	**✔**	**✕**	**✕**
6. Ethical approval provided?	**✕**	**✔**	**✕**	**✕**	**✕**	**✔**	**✔**	**✔**	**✔**	**✔**	**✔**	**✕**	**✕**	**✕**	**✕**	**✔**	**✔**	**✕**	**✔**	**✕**	**✕**	**✕**	**✕**
7. EMS structure described?	**✕**	**✔**	**✔**	**✕**	**✕**	**✔**	**✔**	**✔**	**✔**	**✔**	**✔**	**✕**	**✕**	**✕**	**✔**	**✕**	**✔**	**✕**	**✕**	**✕**	**✔**	**✔**	**✕**
8. Incident clearly described?	**✔**	**✔**	**✔**	**✔**	**✔**	**✔**	**✔**	**✔**	**✔**	**✔**	**✔**	**✕**	**✕**	**✕**	**✔**	**✔**	**✔**	**✕**	**✔**	✔	**✔**	**✔**	**✕**
9. Indications of missing data?	**✕**	**✕**	**✕**	**✕**	**✕**	**✕**	**✔**	**✕**	**✕**	**✔**	**✕**	**✕**	**✕**	**✕**	**✕**	**✕**	**✕**	**✕**	**✕**	**✕**	**✕**	**✕**	**✕**
10. Other limitations discussed?	**✕**	**✕**	**✕**	**✕**	**✕**	**✕**	**✔**	**✕**	**✕**	**✔**	**✕**	**✕**	**✕**	**✕**	**✕**	**✔**	**✔**	**✕**	**✕**	**✕**	**✕**	**✕**	**✕**
11. Study design clearly described?	**✔**	**✔**	**✔**	**✕**	**✔**	**✔**	**✔**	**✔**	**✔**	**✔**	**✔**	**✕**	**✕**	**✕**	**✕**	**✔**	**✔**	**✔**	**✔**	**✕**	**✔**	**✕**	**✕**

*EMS* emergency medical service

### Data extraction

**Fig. 2:**
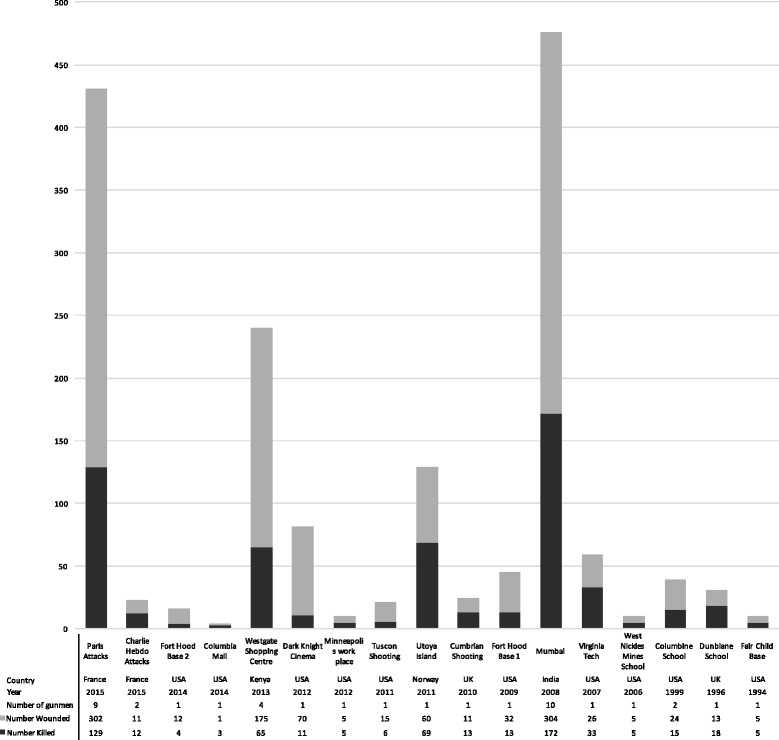
Mass shootings descriptive

The reports described the shooting of 1649 people with 578 deaths at 17 separate events. Fifteen events happened between 2005 and 2015 and include ten single shooter incidents and four multiple shooter attacks. Three events involved concurrent explosions (Fig. 2 and Table 3).

**Table 3:** Data extraction from primary source information

Year of shooting	Author	Mass shooting	Manuscript	Use of TEMS	Scene safety concerns for EMS and delay of treatment	Triage identified as an area for improvement or concern	Type of triage tool used	Support for triage performed by experienced practitioners	Disaster preparedness training important	Communication improvement
2015	Tourtier et al.	Charlie Hebdo	Commentary	N	-	-	-	-	-	-
2015	Hirsch et al.	Paris attacks	Case report	N	Y	-	-	Y	Y	-
2015	Phillipe et al.	Paris attacks	Case report	N	-	-	-	-	Y	Y
2015	Lawrence et al.	Paris attacks	Commentary	Y	Y	N	FTS	Y	-	-
2014	Levy et al.	Columbia Mall	Commentary	Y	Y	-	-	-	-	-
2014	Strommen et al.	Fort Hood Base 2	Case report	N	-	Y	SMART	_	Y	Y
2013	Wachira et al.	Westgate Shopping Centre	Case report	N	Y	Y	-	-	Y	-
2012	Johnson et al.	Dark Knight Cinema	Commentary	N	Y	-	-	-	Y	Y
2012	Koehler et al.	Dark Knight cinema	Case report	N	-	-	-	-	Y	Y
2012	Autrey et al.	Minneapolis Work Place	Case report	Y	N	-	-	-	Y	N
2011	Sollid et al.	Utoya Island	Case report	N	Y	Y	-	Y	Y	Y
2011	Waage et al.	Utoya Island	Case report	N	-	Y	-	-	-	Y
2011	Barr et al.	Tuscon	Commentary	-	-	-	-	-	-	-
2010	Chesterman et al.	Cumbria	Judicial report	Y	Y	N	-	-	Y	Y
2009	Wild et al.	Fort Hood Base 1	Case report	N	Y	Y	LTP	-	-	Y
2008	Roy et al.	Mumbai	Case report	N	Y	N	-	-	-	-
2007	Kaplowitz et al.	Virginia Tech	Case report	-	-	Y	START	-	Y	Y
2007	Timothy et al.	Virginia Tech	Case report	Y	Y	-	START		Y	Y
2006	Ressel R et al.	West Nickles Mines	Case report	-	-	-	-	-	-	-
1999	Mell H et al.	Columbine School	Case report	N	Y	Y	-	-	Y	Y
1996	Cullen, et al.	Dunblane School	Judicial report	N	-	N	-	-	Y	y
1994	Beyersdorf, et al.	Fair Child Base	Case report	N	Y	Y	P	Y	Y	Y
Number of supporting manuscripts				5	12	8		4	14	13

*TEMS* Tactical Emergency Medical Service, *EMS* Emergency Medical Service, *Y* yes, *N* no, - Not addressed in the report, *S* simple triage and rapid treatment (START), *P* physiological, *LTL* local triage protocol, *FTS* Field Triage Score

Fourteen incident sources describe the importance of disaster preparedness planning. Eight stated that triage was an area for improvement and four that the use of experienced practitioners achieved better triage. Thirteen incidents had communication failures. Scene safety was highlighted as an issue for twelve of the mass shootings; five of these supported Tactical Emergency Medical Services (TEMS) (Table 3).

### Epidemiology

Though many organizations have estimated the prevalence of mass shootings, accurate figures are unavailable. In the USA there have been between 62 and 78 mass shooting events between 1983 and 2013 according to the FBI definition; this involves the shooting of between 1023 and 1056 casualties, among whom 507–547 died [2, 55]. A crowd-sourced US website, which refers to an incident with four people shot rather than people killed, reports considerably higher numbers exclusively for the USA; 330 mass shooting events in 2015, 283 in 2014 and 363 in 2013 alone. Although not comprehensively documented there have been a number of devastating attacks recorded in Europe and across other continents.

### Important themes

The Joint Committee to Create a National Policy to Enhance Survivability from Mass Casualty Shooting [56] reported a number of key messages on pre-hospital management of mass shootings summarized by the acronym THREAT, which stands for threat suppression, haemorrhage control, rapid extraction at the scene for assessment by medical providers referring to triage and then finally transport to definitive care.

### Threat suppression and scene safety

At the Utoya Island shooting in 2011, limited interagency communication and a persisting threat by the shooter resulted in EMS staff unable to get onto the island and access casualties for two hours and seven minutes [48] Similarly in the report on the Columbine Shootings of 1999, injured victims were still stranded in the school over two hours after the shooters had committed suicide. Furthermore due to a breakdown in communication a medical team almost came under friendly fire by the Special Weapons And Tactics (SWAT) team [36]. In the UK after the Cumbria shootings in 2010, Chesterman’s report identified interoperability of the police and ambulance as a “highly significant issue leading to extensive delays of ambulances reaching patients and police being left to transport the injured” [57]. This report concluded that “it would be reasonable for the public to expect the ambulance service to attend scenes where there is a residual risk” even though the police service will not be able to guarantee the safety of the staff [16].

Tactical Emergency Medical Support (TEMS) refers to “a non-military medical emergency service modified for the realities of the tactical environment” [58]. TEMS range from personnel trained to work in “hot” or “warm” zones, to those that operate in the safe areas but have specialist training within law enforcement in order to facilitate a greater level of co-operation between the two agencies in a time of extreme pressure. The concept of EMS operating in hazardous environments is changing [42, 59].

Supporting evidence found in this review for the effectiveness of TEMS is mostly anecdotal and from case reports [11, 15, 33]. However, a comprehensive outcome-based report from Finland shows that TEMS integrated into daily EMS could provide effective treatment in law enforcement operations only in safe working zones [60].

A recent concept by Autrey et al. in Minnesota called 3 Echo, “Enter, Evaluate and Evacuate”, looks at a streamlined method for extraction and administration of life-saving haemorrhage control to victims of active shooters. The events of the 27 September 2012 in Minneapolis put “3 Echo” into action [11]. The implementation of this framework on the day led to the safe evacuation of all critically ill patients in a time, to the knowledge of the authors, unmatched for events of this kind. The main principles of this framework are improved communication between services, a shared goal of early identification of casualties by the first wave of law enforcement and establishing safety corridors for evacuation. Instead of clearing large geographical areas such as an entire school, corridors of safety are established as a means of early access to and evacuation of casualties even before the attack has ended.

### Haemorrhage control

Haemorrhage is the leading preventable cause of death in trauma and causes 30–40 % of fatalities [61]. The primary principle of the Hartford Consensus was that nobody should die from uncontrolled bleeding [26]. Pre-hospital medical management at mass shootings is addressed in military settings by Tactical Casualty Combat Care (TCCC) guidelines prioritising the control of catastrophic haemorrhage [23, 24, 27, 51]. These principles are transportable to civilian response where TEMS or trained police officers need to be trained to administer care in the hyper-acute setting [42]. In Paris a damage control strategy was employed, which focused on maintaining a mean arterial pressure >60 mmhg using tourniquets, administration of tranexamic acid and prevention of hypothermia [23]. Damage control resuscitation aims to correct the triad of coagulopathy, acidosis and hypothermia, which can exacerbate bleeding [62]. Remote or “Ground zero” damage control in a pre-hospital setting includes reducing haemorrhage with tourniquets, haemostatic dressings and pelvic binders, minimising hypothermia by reducing exposure, and using thermal blankets and fluid resuscitation with red blood cell transfusion [63]; however, the current literature provides limited evidence for the use of pre-hospital blood products in a civilian setting [64].

### Assessment by medical providers

Triage at mass shooting incidents can be complicated by the ongoing threat and the mechanism of injury [36]. Superficial wounds with minimal visible bleeding may be overlying extensive internal damage and internal haemorrhage and the same is true for the reverse; severe surface injuries do not always indicate extensive damage of internal organs [10]. The inherent challenges with gunshot wound triage are exacerbated by the volume of patients that can be seen in a mass shooting and particular problems are associated with paediatric incidents [17]. Physiological triage utilises vital signs, can be done quickly and requires little experience. In contrast, an anatomical approach relies on accurate diagnosis of injuries and may not be possible without imaging or surgery [65]. Currently the methods of triage for mass shootings are not validated by reliable evidence [66]. In many of the papers included in this review, triage was identified as an area of potential improvement or concern [13, 30, 48, 49, 53, 54].

Triage is a healthcare resource allocation system used when available resources are potentially insufficient to address the needs of patients at a mass casualty event. It attempts to provide the maximum benefit for the most casualties with the available resources [67, 68]. The mass shootings reported in the results section used a mixture of the START triage system [30], local physiological-based triage systems [54], unspecified methods of triage by experienced medical staff [48], an anatomic site of bullet entry triage methods [53] and, in some cases, moving patients rapidly to nearby hospitals where triage occurred after arrival [46].

At the Virgina Tech shooting in 2007 the rate of over-triage was 69 %, measured as patients assigned priority 1 at triage, who actually had an Injury Severity Score (ISS) of less than 15. High levels of over-triage may deprive severely ill patients of the resources needed to treat them, and also put pressure on the surge capacity of the trauma hospital. The Fair Child Massacre also noted a high over-triage rate and expressed the need for a triage tool that could address this problem [13]. Both the Fair Child and Columbine School massacres recorded the issue of uninjured ambulatory patients, processed as “priority 3 delayed”. This occupied the triage resources and prevented the identification of the severely injured. The Fort Hood report concludes that a lack of scene safety, chaotic triage organisation and communication failures led to fear amongst the emergency staff, which compromised triage and led to the inappropriate or under-triage of several patients [54]. A second attack on this base in 2014 reported similar triage issues, noting that ongoing clinical reassessment was critical [49].

The benefit of using experienced pre-hospital experts who have seen large numbers of critically ill patients was reported by those at Utoya [48]. Since this incident a national manual for mass casualty triage has been created in Norway [69]. Two other incidents reported the benefits of having senior surgeons play a pivotal role in pre-hospital triage; the terrorist attacks in Jerusalem and the Dark Knight cinema shooting in Aurora Colorado [32, 45]. Furthermore both also supported the concept of keeping initial triage simple [31, 54].

Allocation of patients to appropriate services and maintaining good flow in these hospitals is important to avoid saturation of the receiving hospitals. Poor communication at the incident can lead to errors in patient transport, delivering patients to inferiorly equipped hospitals [52]. It has been shown that admission to a level-1 trauma centre reduces mortality for the most severe trauma patients [70]. At the Westgate shooting patient flow was maintained by the increased availability of porters and by creating of a one-direction system, keeping the emergency department clear for arriving casualties [53].

### Rapid extraction and disaster preparedness

Major incident planning and preparedness focuses on the cross-service organisation of infrastructure to facilitate good communication, command and control, safety at the scene, accurate triage and the transport of patients in unpredictable environments [65].

The reviewed literature links disaster preparedness to a successful response. A lack of familiarity and unpreparedness predisposes to confusion and delay [11, 13, 23, 24, 28, 30, 32, 36, 49]. The low mortality rate in the Virginia Tech shooting of 2007 was credited to the training and collaborative planning that had been a focus since the 11 September 2001 attacks [30]. After the Aurora Dark Knight cinema shootings of 2012, the authors agreed that these incidents cannot be rehearsed too frequently and supported the idea of tabletop exercises [32]. The responders to the Minneapolis workplace shooting in 2012 had undertaken a 12-hour training curriculum, which involved tabletop exercises and hands-on, walk-through scenarios, which were highly realistic involving replica weapons and ammunition. The 3 Echo protocol training resulted in triage and evacuation of patients with reported unprecedented efficiency in a subsequent mass shooting emergency response. The interagency exercises that occurred on the morning of the attacks in Paris were considered a key factor in the success of treatment; the large hospital network coordinated by Assistance Publique-Hôpitaux de Paris was also highlighted as a strength [23].

Simple measures to ensure preparedness such as having dedicated medical equipment stores for major incidents and the value of equipment such as lightweight stretchers for the transport and tracking of injured patients have been recommended [11, 48, 71]. Targeted training can have a significant impact on perceptions of EMS preparedness. One study demonstrated that 41 % of responders felt prepared to respond to an active shooting incident before training, increasing to 89 % after tactical awareness training [29].

Communication failures are a consistent feature of post-major incident reports and at mass shootings these failures can have detrimental impacts [13, 16, 19, 30, 48, 49, 54]. At Fort Hood two patients were transported to hospitals with inadequate trauma facilities due to loss of communication [54]. Columbine in 1999 experienced problems with radio “dead zones” and a miscommunication meant that at one point it was believed that eight, not two, shooters were active in the school [36]. False alarms at the Virginia Tech shooting in 2007 caused ambulances to be evacuated unnecessarily [50]. Failure of the Norwegian Acute Medical Information System at logging communications caused issues in tracking patient pathways and destination hospitals. Tracking problems were also reported by Kaplowitz at Virgina Tech 2007 [30, 48]. Having backup communications equipment, pre-arranged disaster channels for communication and establishing communication protocols with public area authorities, such as train stations, schools and large buildings, has been suggested as a means to mitigate these issues [11, 36, 54].

## Discussion

This systematic literature review identified the need for integration of tactical emergency medical services [11], improved cross-service education on effective haemorrhage control [27], the need for early effective triage by senior clinicians and the need for regular mass casualty incident simulation [5, 23].

Scene safety is a unique challenge at mass shooting incidents. Active shooters produce a dangerous and unpredictable environment where the number of casualties can keep increasing and the safety of the emergency staff is compromised. Poor scene safety delays EMS staff treating patients, and contributes to failures of major incident organisation and communication [13, 16, 24, 28, 30, 32, 34–36, 46, 52–54]. Aggressive entry into unsafe areas containing casualties has been promoted in consensus statements [24].

The traditional hierarchy of major incident pre-hospital medical management is for triage, treatment and then transport [65]. This method has been challenged recently to put catastrophic haemorrhage treatment as the top priority and additionally to create a provision for unwounded survivors to be moved to a separate survivor centre to reduce the burden on hospitals receiving the seriously injured. The UK has recently undertaken these changes to its triage system following advice from the Royal Centre for Defence Medicine after studies completed during the Iraq and Afghanistan conflicts, and due to recommendations from the London Bombings Inquiry [72].

The US Federal Emergency Management Agency created a resource document in 2013. It recommended joint planning and training of law enforcement and EMS personnel, practice for rapid treatment and evacuation, the use of tabletop and field exercises and standardised terminology [37]. Joint training and preparation for major incidents more generally in the UK is the focus of the Joint Emergency Services Interoperability Programme, which aims to improve communication, situational awareness and co-operation of the three emergency services through joint training and exercise [73].

### Limitations

Drawing direct comparison between similar incidents is sometimes difficult and anecdotal evidence in individual reports means the authority of the recommendations were uncertain in some cases. There are no suitable validated quality appraisal tools so a non-validated checklist was used. Non-English literature was excluded and there was no meta-analysis of data. Government papers and inquiries focus on either the operation of law enforcement on the motives of the shooter, and less emphasis is given to the emergency medical response [16, 19].

Uniform and comprehensive reporting of mass casualty shootings is required, along with a dedicated recording database for the occurrence of these incidents outside the USA [5]. Epidemiology in this global phenomenon is incomplete; clinical experience from shootings in many countries has not been published, including high profile events like the Sousse attacks in Tunisia 2015. This review is based on low-quality retrospective case report analysis, and anecdotal expert opinion. There is a need to design high-quality reporting and research in this area. There is likely to be reporting bias in this topic due to its sensitivity, and some documentation may be classified due to involvement of counter-terrorism agencies. Strengths of this review include having two authors with extensive pre-hospital experience, the use of a systematic approach and the incorporation of additional information found in the grey literature.

## Conclusion

Mass casualty civilian shootings represent an infrequent but recurring challenge to emergency services with distinct management challenges. This systematic review identified 14 case reports and 33 further documents, using a systematic search strategy detailing the pre-hospital medical response to mass shooting of 1649 people and collected the advice and lessons from the experiences of those involved.

Threat suppression and inner-cordon medical interventions may be harmonised by TEMS, providing a platform for better education, training and on-scene communication. Haemorrhage control equipment should be widely available along with inter-service and general public education on effective bleeding control in penetrating trauma. The use and effectiveness of particular triage tools is not well-established but the use of senior medical providers to carry out initial assessment appears to increase effective casualty management. Regular and specific multi-disciplinary and multi-service preparedness exercises are essential to ensure a successful response, and should include local public and private authorities in exercises involving schools and other public areas.

The uniform and comprehensive reporting of major incidents, requiring an agreed nomenclature, needs to be improved, and will facilitate progression in research [74]. A reporting tool for this purpose has recently been created [5].

## Abbreviations

EMS: Emergency Medical Service
FBI: Federal Bureau of Intelligence
SWAT: Special Weapons and Tactics
TEMS: Tactical Emergency Medical Service
