# Erratum to: Pre-hospital management of mass casualty civilian shootings: a systematic literature review

**DOI:** 10.1186/s13054-017-1663-8

**Published:** 2017-04-13

**Authors:** Conor D. A. Turner, David J. Lockey, Marius Rehn

**Affiliations:** 1 Barts and The London School of Medicine and Dentistry, Queen Mary University London, Garrod Building, Turner Street, Whitechapel, London, E1 2AD UK; 2 London’s Air Ambulance, Barts Health Trust, London, UK; 3 The Norwegian Air Ambulance Foundation, Drøbak, Norway; 4 Department of Health Studies, University of Stavanger, Stavanger, Norway

## Erratum

Unfortunately this article [1] was published with an error. Reference 33 was incorrectly identified as “Lawrence D. Tactical emergency medicine : lessons from Paris marauding terrorist attack. Crit. Care 2016; 20:37”. This should have been “Service médical du RAID. Tactical emergency medicine : lessons from Paris marauding terrorist attack. Crit. Care 2016; 20:37”. Reference 33 is cited in tables and text.
